# Multi‐TPS institutional surveillance of VMAT plan complexity and PSQA results over three years

**DOI:** 10.1002/acm2.70691

**Published:** 2026-07-06

**Authors:** Sotiri Stathakis, Ara Alexandrian, Mason Heath

**Affiliations:** ^1^ Department of Medical Physics and Radiation Oncology Mary Bird Perkins Cancer Center Baton Rouge Louisiana USA

**Keywords:** dosimetrist variability, MCSv, Monaco, MOSAIQ, MU factor, oncology information system, Pinnacle, plan complexity, quality assurance, treatment planning system, VMAT

## Abstract

**Background:**

Running VMAT plan‐complexity surveillance directly inside an oncology information system (OIS) makes it practical to monitor plan quality across a large, multi‐linac program. Complexity metrics are increasingly used to anticipate patient‐specific QA (PSQA) outcomes, but few studies span multiple years or tie complexity monitoring to the OIS at scale, which leaves published benchmarks hard to generalize and prospective monitoring slow to enter routine practice.

**Purpose:**

To describe an automated Python pipeline integrated with the MOSAIQ OIS for computing eight established VMAT plan complexity metrics from DICOM‐RT files at scale, and to characterize the resulting distributions across a three‐year institutional VMAT program stratified by treatment planning system (TPS), anatomic site, dosimetrist, and calendar year, with correlation to matched PSQA outcomes.

**Methods:**

All clinically approved VMAT plans from March 2023 to March 2026 were identified via MOSAIQ OIS (v2.6, Elekta AB) at a multisite comprehensive cancer center. A purpose‐built Python tool queried the MOSAIQ SQL Server database and parsed DICOM‐RT plan files to compute MU Factor, mean aperture area (MAA), mean leaf gap (MLG), aperture irregularity (AI), small aperture scores (SASs) at 5 and 10 mm, modulation complexity score for VMAT (MCSv), and Modulation Index Total (MITotal). Plans were stratified by TPS (Pinnacle3 vs. Monaco), anatomic site group (11 groups), dosimetrist, and year. Mann–Whitney U and Kruskal–Wallis tests were applied for group comparisons. PSQA pass rates (3%/2 mm global gamma, ≥95% threshold) were extracted from MOSAIQ and matched to complexity data for 3402 of 3751 plans (90.7%); Spearman rank correlations quantified complexity–pass‐rate associations.

**Results:**

The institutional median (IQR) MU Factor was 2.96 (2.26–4.00) MU/cGy and MCSv was 0.966 (0.955–0.978). Monaco plans showed significantly higher MU Factors (+32%, *p* < 0.001), narrower apertures (MLG −16%), and higher MCSv (+1.0%) than Pinnacle3 plans. Pelvis and head and neck plans had the highest median MU Factors (3.77 and 3.40 MU/cGy); Breast plans were most efficient (2.10 MU/cGy). Institutional MU Factor rose 20% over three years. Of 3402 PSQA‐matched plans, the mean gamma pass rate was 98.7% (median 99.6%); 115 (3.4%) fell below 95%. All complexity metrics correlated significantly with pass rate (all *p* < 0.001); AI (ρ = −0.358) and MCSv (ρ = +0.336) were the strongest predictors. Monaco plans achieved a higher mean PSQA pass rate than Pinnacle3 (99.0% vs. 98.5–98.6%) despite greater modulation. Significant linac‐level variation in pass rates was observed (97.3%–99.2%), not fully explained by plan complexity.

**Conclusions:**

OIS‐integrated VMAT complexity and PSQA surveillance is feasible at scale and yields clinically useful information. AI and MCSv were the strongest complexity‐based predictors of PSQA outcome. The TPS‐, site‐, and linac‐specific complexity profiles we observed argue for risk‐stratified PSQA design. The pipeline is readily transferable to other MOSAIQ‐based programs for prospective plan‐quality monitoring.

## 1 Introduction

Volumetric modulated arc therapy (VMAT), introduced by Otto in 2008,^1^ is now the standard way to deliver intensity‐modulated radiation therapy. By simultaneously modulating multileaf collimator (MLC) positions, gantry speed, and dose rate during continuous arc delivery, VMAT achieves highly conformal dose distributions with improved normal tissue sparing and shorter treatment delivery times compared with static‐gantry IMRT.^2^, ^3^

The same flexibility introduces treatment plan complexity—characterized by irregular aperture shapes, narrow leaf gaps, rapid leaf travel, and elevated monitor unit (MU) counts relative to prescribed dose—that can reduce agreement between planned and delivered dose distributions.^4^, ^5^ Quantitative complexity metrics have therefore emerged as tools for prospectively identifying plans at elevated risk of patient‐specific quality assurance (PSQA) failure. Key validated metrics include the MU Factor,^4^ modulation complexity score for VMAT (MCSv),^5^ small aperture score (SAS),^4^ and modulation index total (MITotal).^6^ Despite their utility, most published analyses are limited by small sample sizes, single‐site or single‐disease designs, and cross‐sectional scope,^7^ and direct TPS‐to‐TPS platform comparisons on the same linac fleet remain scarce.^8^, ^9^

This technical note describes an automated Python pipeline integrated with the MOSAIQ OIS that computes eight complexity metrics for every approved VMAT plan, with results stratified by TPS, anatomic site, dosimetrist, and calendar year, and correlated with matched PSQA outcomes across 3751 plans over three years.

## 2 METHODS AND MATERIALS

### 2.1 Patient cohort and data source

All clinically approved VMAT plans created between March 2023 and March 2026 at a multisite comprehensive cancer center operating Elekta Agility, Versa HD, and Harmony linacs were identified via MOSAIQ OIS (v2.6, Elekta AB). An MR‐guided linac dedicated to MR‐only treatments was excluded as it does not perform standard VMAT. A retrospective analysis of plan dosimetric data required no IRB review under institutional policy.

### 2.2 Automated pipeline

**FIGURE 1:**
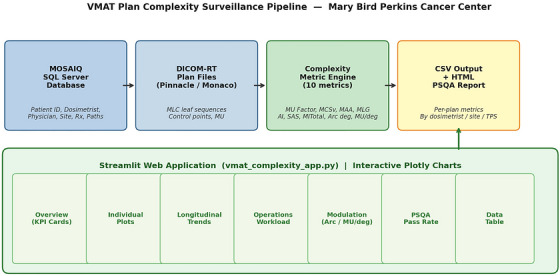
Schematic overview of the automated VMAT plan complexity surveillance pipeline. The MOSAIQ SQL Server database is queried to identify all approved VMAT plans; DICOM‐RT files are parsed by the Python engine to compute ten complexity metrics, stored as CSV with an HTML PSQA report. The paired Streamlit/Plotly web application provides seven interactive tabs: Overview, individual plots, longitudinal trends, operations, modulation, PSQA pass rate, and data table.

A purpose‐built Python application queried the MOSAIQ SQL Server database (pyodbc) to extract treatment course data and DICOM‐RT plan file paths; pydicom was used for file parsing. A parallelized computation engine enabled multi‐threaded metric calculation. Results were visualized through a browser‐based Streamlit/Plotly web application providing seven interactive tabs (Figure 1).

### 2.3 Complexity metrics

Eight metrics were computed per plan:^4^, ^5^, ^6^ MU Factor (MU/cGy); mean aperture area (MAA, mm^2^); mean leaf gap (MLG, mm); aperture irregularity (AI); SASs at 5 and 10 mm thresholds (SAS_5__m__m_, SAS_10__m__m_); MCSv; and MITotal. Treatment site labels were mapped to eleven anatomic groups using exact‐ and substring‐match rules with manual verification.

### 2.4 Statistical analysis and PSQA

Descriptive statistics and Mann–Whitney U or Kruskal–Wallis tests (*p* < 0.05; Python 3.11, SciPy) were used for group comparisons. PSQA was performed using MapCHECK detector arrays (Sun Nuclear) per AAPM TG‐218 protocols.^10^ The MapCHECK array was calibrated annually; in addition, prior to each PSQA measurement session a 200 cGy reference dose was delivered under reference conditions to correct for daily linac output variation. Gamma analysis used 3%/2 mm global criteria with a 10% dose threshold; ≥95% point‐pass defined a passing plan. The composite dose from all VMAT arcs in each plan was compared against a single MapCHECK measurement; no beam‐by‐beam gamma analysis was performed. Because the recorded gamma pass rates are archival, re‐evaluation under stricter gamma criteria (e.g., 2%/2 mm) was not feasible. Of 3751 plans, 3402 (90.7%) had matched PSQA records. Spearman rank correlations (ρ) quantified complexity–pass‐rate associations.

## 3 RESULTS

### 3.1 Cohort and overall distributions

The cohort included 3751 plans from 20 dosimetrists and 15 radiation oncologists (Table 1). Pinnacle3 generated 2610 plans (69.6%); Monaco generated 1138 (30.3%). The most common site groups were Prostate (17.0%), Thorax (14.8%), and Pelvis (14.1%). Overall metric distributions are presented in Table 2; the institutional median MU Factor was 2.96 (IQR 2.26–4.00) MU/cGy and MCSv was 0.966 (IQR 0.955–0.978).

**TABLE 1:** Cohort characteristics.

Characteristic	Value	Notes
Total plans	3751	VMAT only
Date range	March 2023–March 2026	3‐year institutional cohort
Dosimetrists	20	All active planners included
Radiation oncologists	15	
Treatment planning systems	Pinnacle3 (*n* = 2610) / Monaco (*n* = 1138)	3 plans from other TPS excluded
Anatomic site groups (11)	Prostate, head and neck, thorax, pelvis, breast, brain, spine, abdomen, extremity, skin, other	
PSQA‐matched plans	3402 (90.7%)	3%/2 mm global gamma, ≥95% threshold

Abbreviations: PSQA, patient‐specific quality assurance; TPS, treatment planning system; VMAT, volumetric modulated arc therapy.

**TABLE 2:** Complexity metric summary: Overall cohort and Pinnacle3 versus Monaco comparison.

Metric	Overall median [IQR]	Pinnacle3 (*n* = 2610) median [IQR]	Monaco (*n* = 1138) median [IQR]	Δ vs. P3	*p* value	Sig.
MU Factor (MU/cGy)	2.96 [2.26–4.00]	2.76 [2.14–3.57]	3.63 [2.70–4.87]	+32%	<0.001	^***^
MAA (mm^2^)	3971 [2433–5839]	4328 [2643–6218]	3380 [2025–5019]	−22%	<0.001	^***^
MLG (mm)	33.00 [27.63–39.19]	34.96 [29.33–41.31]	29.20 [24.42–34.10]	−16%	<0.001	^***^
AI	1.42 [1.07–2.02]	1.39 [1.05–1.94]	1.50 [1.11–2.23]	+8%	<0.001	^***^
SAS10mm	0.000 [0.000–0.005]	0.000 [0.000–0.000]	0.000 [0.000–0.005]	↑ Monaco	<0.001	^***^
MCSv	0.966 [0.955–0.978]	0.963 [0.951–0.976]	0.973 [0.963–0.981]	+1.0%	<0.001	^***^
MITotal	0.149 [0.132–0.174]	0.154 [0.140–0.181]	0.136 [0.117–0.159]	−12%	<0.001	^***^

*Note*: Values are median [IQR]. Mann–Whitney U test for the TPS comparison.

Abbreviations: AI, aperture irregularity; MAA, mean aperture area; MCSv, modulation complexity score for VMAT; MITotal, modulation index total; MLG, mean leaf gap; P3, Pinnacle3.

*** *p* < 0.001. SAS5mm omitted (near‐zero throughout).

### 3.2 TPS comparison

**FIGURE 2:**
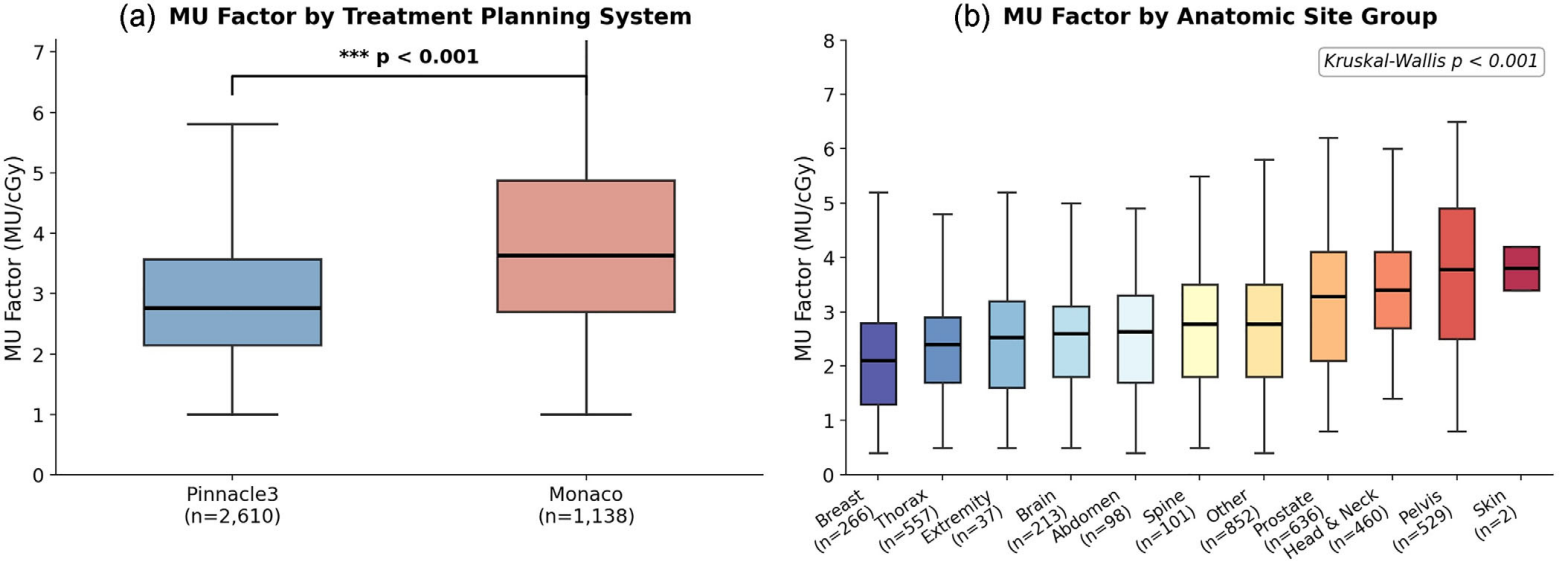
Box plots of MU Factor stratified by (a) treatment planning system (TPS) and (b) anatomic site group (ordered ascending by median). *** *p* < 0.001 (Mann–Whitney U). RdYlBu color gradient reflects complexity rank.

**FIGURE 3:**
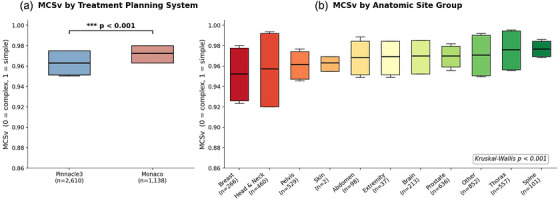
Box plots of MCSv stratified by (a) TPS and (b) anatomic site group. Lower MCSv indicates greater complexity. Head and neck plans show the widest spread (SD 0.036).

All eight metrics differed significantly between TPS platforms (Table 2; all *p* < 0.001). Monaco plans required 32% more MU per cGy (median 3.63 vs. 2.76), had smaller aperture areas (MAA −22%), narrower leaf gaps (MLG −16%), and higher aperture irregularity (AI +8%), yet displayed higher MCSv (+1.0%), indicating smoother arc trajectories. MITotal was 12% lower for Monaco (Figures 2, 3).

### 3.3 Anatomic site and temporal variation

**FIGURE 4:**
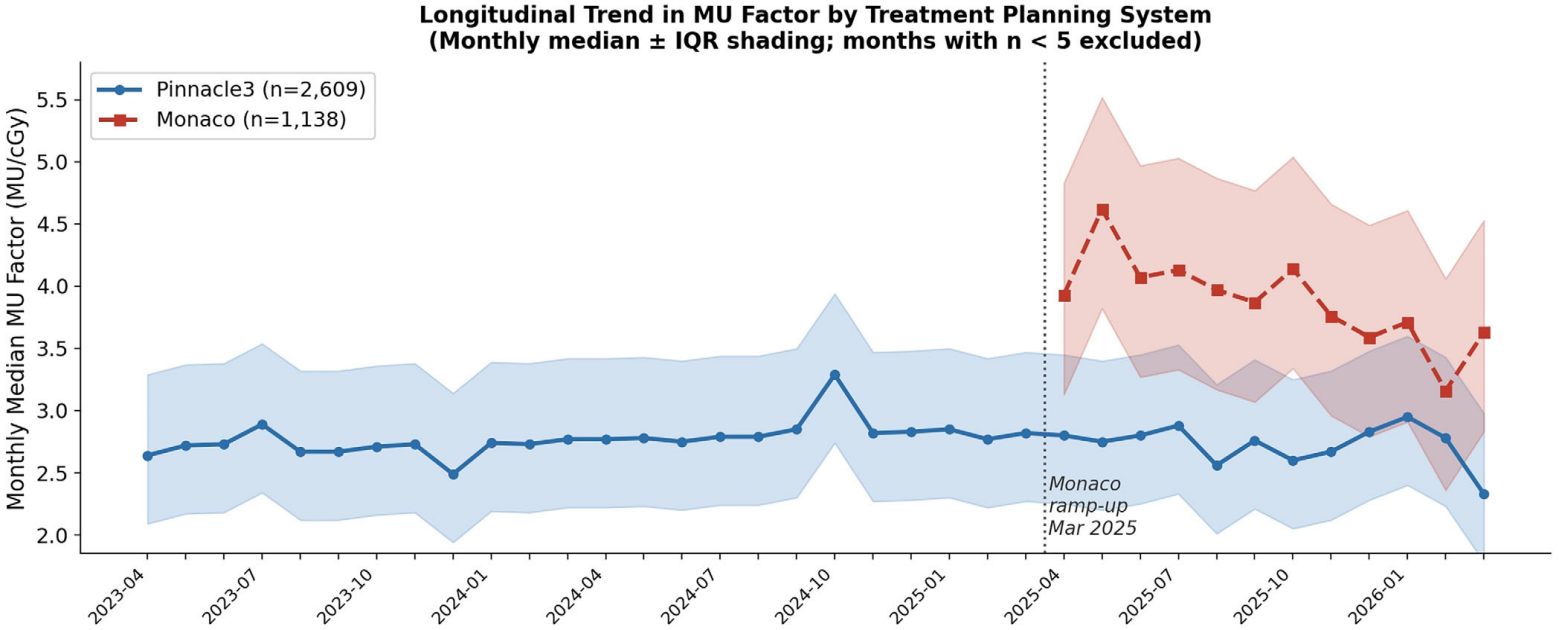
Longitudinal monthly median MU Factor (March 2023–March 2026) by TPS. Blue solid = Pinnacle3; red dashed = Monaco. Shaded bands = IQR. Dotted vertical line marks Monaco clinical ramp‐up (March 2025).

Significant cross‐site differences were observed for all metrics (Kruskal–Wallis *p* < 0.001). Pelvis and head and neck plans had the highest median MU Factors (3.77 and 3.40 MU/cGy); Breast plans were most efficient (2.10 MU/cGy). Head and neck showed the widest MCSv spread (SD 0.036). Institutional median MU Factor rose 20% over three years (2.67 in 2023 to 3.21 MU/cGy in 2026; Figure 4), but this institutional‐level increase coincided with the clinical introduction of Monaco in March 2025: within‐platform Pinnacle3 and Monaco medians remained comparatively stable across their respective active periods, indicating that the institutional rise reflects a shift in TPS case mix rather than a within‐platform trend in planning practice.

### 3.4 PSQA correlations and pass rates

**FIGURE 5:**
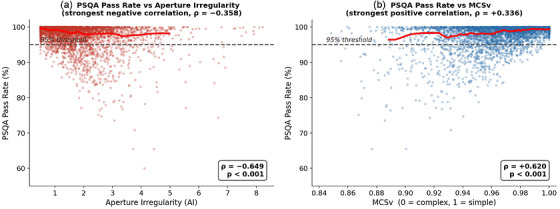
PSQA pass rate versus (a) AI (ρ = −0.358, strongest negative) and (b) MCSv (ρ = +0.336, strongest positive). Red curves = running‐median smooth. Dashed line = 95% threshold (*n* = 3402).

**FIGURE 6:**
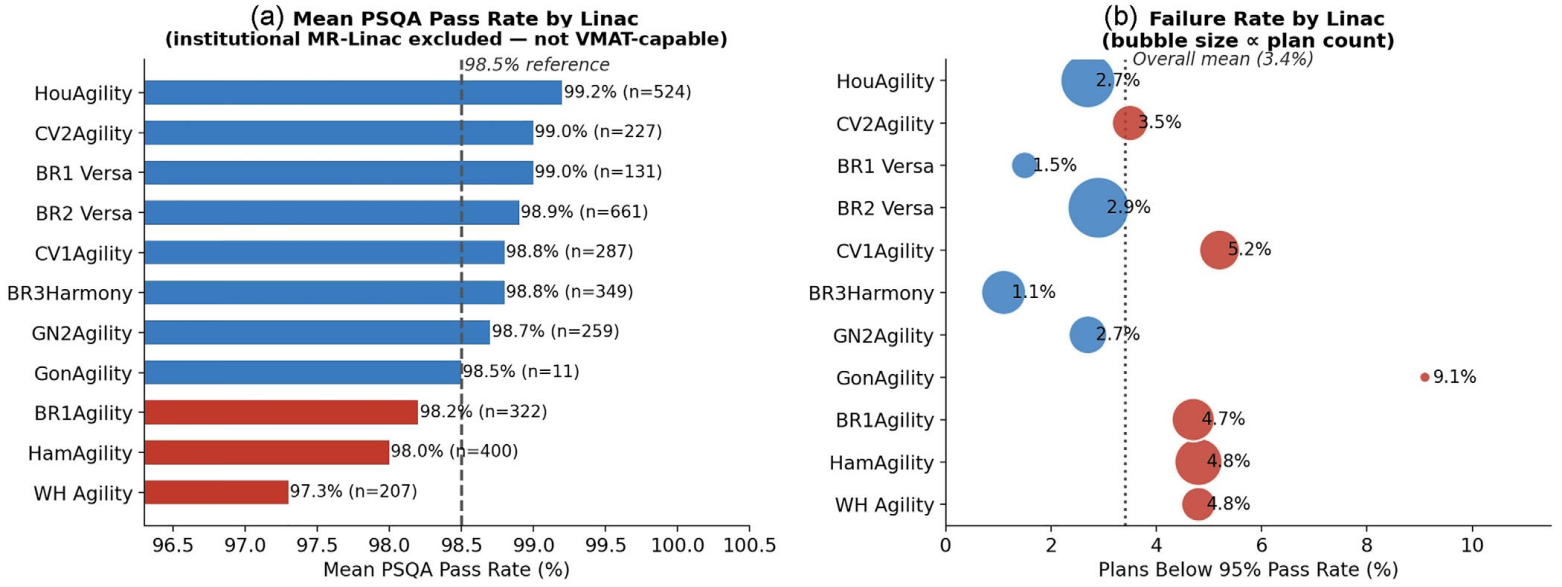
PSQA pass rates by linac (the institutional MR‐guided linac excluded). (a) Mean pass rate; bars red if below 98.5% reference. (b) Failure rate (% plans < 95%); bubble size ∝ plan count. Dotted line = overall cohort mean (3.4%).

Of 3402 PSQA‐matched plans, the mean pass rate was 98.7% (median 99.6%); 115 (3.4%) fell below 95% (Table 3; Figure 5). All complexity metrics correlated significantly with pass rate (all *p* < 0.001). AI was the strongest negative predictor (ρ = −0.358); MCSv the strongest positive (ρ = +0.336). Monaco plans achieved higher mean pass rates than Pinnacle3 (99.0% vs. 98.5–98.6%) despite greater modulation. Linac‐level pass rates ranged from 97.3% to 99.2% (Figure 6) and were not fully explained by plan complexity differences across machines.

**TABLE 3:** PSQA summary.

A. Spearman correlations — PSQA pass rate vs. complexity metrics (*n* = 3402)						
Metric	ρ	*p* value	Interpretation			
AI	−0.358	<0.001	Strongest negative			
MCSv	+0.336	<0.001	Strongest positive			
MAA	−0.278	<0.001				
MU Factor	−0.256	<0.001				
MU/Degree	+0.189	<0.001				
Arc Degrees	−0.178	<0.001				
MITotal	+0.114	<0.001	Weakest			

B. PSQA pass rates by site group and TPS						
Site/TPS	*n*	Mean PR (%)	Med PR (%)	*n* < 95%	Fail (%)	Note
Head and neck	428	98.3	99.2	19	4.4	Lowest pass rate
Thorax	519	98.4	99.9	14	2.7	
Pelvis	484	98.6	99.4	17	3.5	
Brain	197	98.7	99.7	4	2.0	
Breast	239	98.7	99.6	15	6.3	Highest failure rate
Other	752	98.7	99.6	33	4.4	
Abdomen	86	98.9	99.9	2	2.3	
Prostate	577	99.0	99.7	9	1.6	
Spine	89	99.1	99.8	1	1.1	Highest pass rate
Pinnacle3 16.2.x	830	98.6	99.2	—	—	
Pinnacle3 18.0.1	1670	98.5	99.6	—	—	
Monaco 6.2.2.0	896	99.0	99.8	—	—	Best pass rate

Abbreviations: Fail, % of plans below the 95% gamma threshold. Site section sorted ascending by mean PR; PR, pass rate; ρ, Spearman rank correlation; all *p* < 0.001.

## 4 DISCUSSION

This Technical Note reports OIS‐integrated VMAT complexity surveillance across 3751 plans and 20 dosimetrists, to our knowledge the largest such institutional cohort published so far. Because both planning systems drew on the same linac fleet, the 32% higher MU Factor for Monaco reflects the planning system rather than the hardware: Monaco's Monte Carlo engine needs tighter apertures to model tissue heterogeneity. The higher MCSv and lower MITotal for Monaco point to more evenly distributed leaf travel, which produces individually deliverable arc segments and, perhaps surprisingly, a higher mean PSQA pass rate even though the plans are more modulated overall. A center weighing a move to Monaco should not read its higher MU Factors as a QA liability.

AI (ρ = −0.358) was a stronger predictor of PSQA outcome than MCSv, which is worth noting given how much attention MCSv has drawn in the literature.^4^, ^7^ Irregular apertures magnify the dose impact of MLC positioning errors at many leaf tips at once—a concern that is especially relevant for the Elekta Agility MLC. Linac‐level pass rates still varied (97.3%–99.2%) after we accounted for case mix, which points to machine‐specific contributors such as MLC calibration drift and output stability and argues for linac‐stratified thresholds when stratifying risk. The 20% three‐year rise in institutional MU Factor is largely an artifact of the March 2025 Monaco introduction rather than a within‐platform trend in planning practice. This is the practical case for TPS‐stratified longitudinal monitoring: institutional aggregates can be driven by shifts in TPS case mix and can mask otherwise stable planner behavior.

Limitations include modest Spearman correlations (maximum |ρ| = 0.358), possible case‐mix confounding in inter‐dosimetrist comparisons, restriction to an Elekta linac fleet, and institutional PSQA criteria (3%/2 mm global) that may differ from other programs. TPS, machine, and treatment site assignments are partially confounded in this single‐institution observational cohort: certain sites were preferentially planned on Monaco after the 2025 rollout, and TPS‐to‐linac pairing was not random. Reported TPS effects should therefore be interpreted as joint effects within the institutional case‐mix rather than isolated platform‐level effects. Beam‐by‐beam gamma analysis was not performed because PSQA records are archival composite‐dose comparisons; prospective beam‐resolved acquisition and stricter gamma criteria are planned for ongoing surveillance.

## 5 CONCLUSION

Automated, OIS‐integrated VMAT complexity surveillance across 3751 plans identifies AI and MCSv as the strongest PSQA predictors and shows that Monaco produces higher MU Factors yet better PSQA outcomes. The 20% institutional rise in median MU Factor over three years tracks the clinical introduction of Monaco, not a change in planning practice—which is why we monitor complexity separately for each TPS. The Python pipeline is open source and transfers readily to other MOSAIQ‐based programs for prospective, risk‐stratified QA monitoring.

## AUTHOR CONTRIBUTIONS

**Sotiri Stathakis**: Conceptualization; methodology; software; data curation; writing—original draft; supervision. **Ara Alexandrian**: Data curation; validation; writing—review and editing. **Mason Heath**: Formal analysis; visualization; writing—review and editing.

## CONFLICT OF INTEREST STATEMENT

The authors declare no conflicts of interest.

## ETHICAL APPROVAL

Retrospective analysis of plan dosimetric data; no IRB review was required under institutional policy.

## ARTIFICIAL INTELLIGENCE DISCLOSURE

AI‐assisted writing tools were used for language editing during manuscript preparation. All scientific content, analysis, and conclusions were generated and verified by the authors.

## Data Availability

The Python analysis pipeline is available from the corresponding author upon reasonable request.
